# Evaluation of Antidepressant Activity of Capsaicin Nanoemulsion in Nicotine Withdrawal-Induced Depression in Mice

**DOI:** 10.3390/brainsci13121668

**Published:** 2023-12-01

**Authors:** Naveen Kumar Krishnamoorthy, Tamsheel Fatima Roohi, Muthukumar Serva Peddha, Nabeel Kinattingal, Shahid Ud Din Wani, Kamsagara Linganna Krishna, Faiyaz Shakeel, Seema Mehdi

**Affiliations:** 1 Department of Pharmacology, JSS College of Pharmacy, JSS Academy of Higher Education and Research, Mysuru 570015, India; hbnaveen2016@gmail.com (N.K.K.); tamsheelfatimaroohi22@gmail.com (T.F.R.); nabeel.al.makkawi@gmail.com (N.K.); kkrishna@jssuni.edu.in (K.L.K.); 2 Department of Biochemistry, CSIR-Central Food Technological Research Institute (CSIR-CFTRI), Mysuru 570020, India; muthukumar@cftri.res.in; 3 Department of Pharmaceutical Sciences, School of Applied Sciences and Technology, University of Kashmir, Srinagar 190006, India; shahidpharma2013@gmail.com; 4 Department of Pharmaceutics, College of Pharmacy, King Saud University, P.O. Box 2457, Riyadh 11451, Saudi Arabia

**Keywords:** depression, capsaicin, nanoemulsion, inflammation, nicotine withdrawal

## Abstract

Depression is a low-energy condition that has an impact on a person’s thoughts, actions, propensities, emotional state, and sense of wellbeing. According to the World Health Organization (WHO), 5% of adults are depressed. Individuals who are depressed are commonly prescribed antidepressants, and sometimes, individuals may have other psychiatric conditions that share overlapping symptoms with depression. These cooccurring conditions can complicate the diagnostic process, leading to a misdiagnosis and the prescription of antidepressants. Capsaicin (CAP) is a known antidepressant. Hence, this study aimed to assess the antidepressant activity of CAP nanoemulsion in nicotine (NC) withdrawal-induced depression in mice. Mice treated with CAP (3 mg/kg) showed reduced immobility in the forced swimming test (FST), tail-suspension test (TST), and open field test (OFT). During the OFT, the animals treated with nanoemulsion (CAP 3 mg/kg) spent less time in the corners than the control animals. Biochemical parameters, such as superoxide dismutase (SOD) and glutathione (GSH), were observed in reduced quantities in the NC withdrawal model (NWM), where they were slightly increased in the high-dose nanoemulsion (CAP 3 mg/kg) compared to the low-dose nanoemulsion (CAP 1 mg/kg). These results suggest that CAP caused antidepressant activity in the NWM via the nanoemulsion.

## 1. Introduction

Millions of people worldwide struggle with depression, an illness that has the potential risk of causing suicidal tendencies that can lead to death. The onset of depression has been observed from childhood to old age. Depression has a significant negative impact on society by eroding hope, aspiration, and occasionally even the will to live, causing widespread morbidity [1]. It has been stated that depression is one of the prevailing psychiatric disorders which has been estimated as the second most significant cause of neurological diseases and disability worldwide [2].

Nicotine (NC) is an addictive component of tobacco and has anxiolytic (anxiety-reducing) and antidepressant effects [3]. NC withdrawal can induce anxiety and depression, making it a potential model for studying these conditions. NC exhibits the dual effects of being both anxiolytic and antidepressant, contributing to its complex interactions with neural pathways. Transient receptor potential vanilloid 1 (TRPV1) receptors are known to play a role in pain perception and stress responses. Chronic pain and stress are often associated with mood disorders. It is possible that TRPV1 receptor activation or modulation could impact stress-related pathways and contribute to mood regulation. TRPV1 receptors are sensitive to capsaicin (CAP), the compound responsible for the spiciness of chili peppers. Activation of TRPV1 receptors by CAP may induce a release of neurotransmitters that could influence mood [4].

TRPV1 channels are found in almost every region of the brain, but they are particularly abundant in the limbic system structures (amygdala and hippocampus). The TRPV1 receptor, classically known for its role in nociception, has emerged as a multifaceted player in various physiological processes. Beyond its function as a pain receptor, TRPV1 is implicated in the modulation of neurotransmitter release, neuroplasticity, and neurotrophic support [5]. In the realm of neurotransmission, TRPV1 activation influences the release of key neurotransmitters such as dopamine, serotonin, and glutamate. The mesolimbic dopamine system, which is associated with reward and motivation, is particularly responsive to TRPV1 modulation. Additionally, the receptor’s presence in serotonergic neurons and its impact on glutamate release further underscore its relevance in the intricate web of neurotransmitter regulation. Moreover, TRPV1 has been linked to neuroplasticity, affecting long-term potentiation and long-term depression—essential processes for learning and memory. The receptor’s influence extends to the neurotrophic domain, where its activation has been associated with the modulation of brain-derived neurotrophic factor, which is a crucial factor in neuronal survival and differentiation [6]. Furthermore, TRPV1’s expression in the hypothalamus implicates it in the regulation of the hypothalamic–pituitary–adrenal (HPA) axis, suggesting a role in stress hormone release. TRPV1 receptors and the endocannabinoid system are intricately connected, and their interplay contributes to the regulation of various physiological processes. The endocannabinoid system consists of cannabinoid receptors, endogenous cannabinoids (endocannabinoids), and enzymes responsible for their synthesis and degradation. Anandamide and 2-arachidonoylglycerol are two well-known endocannabinoids that bind to cannabinoid receptors, including cannabinoid 1 and cannabinoid 2 receptors. TRPV1 receptors—although traditionally associated with responses to heat and pain—are also activated by the endovanilloids, anandamide, and N-arachidonoyl dopamine, establishing a functional link between TRPV1 and the endocannabinoid system [6,7]. These multifaceted interactions highlight the diverse functions of TRPV1 and set the stage for understanding its potential antidepressant mechanisms [7]. According to certain research, the forced swimming test (FST) for rodents can modify the immobility duration by activating TRPV1. CAP, a TRPV1 agonist, reduces depressive-like behavior in mice [7].

CAP is one TRPV1 ligand whose antidepressant and anxiolytic effects have received scant attention. Many types of chili peppers contain CAP, which gives them their spiciness and pungent flavor. TRPV1 channels are found in the central and peripheral nervous systems and are closely linked to neurotransmission. These channels, known for their role in detecting and responding to heat and CAP, have been increasingly recognized for their involvement in various physiological and pathological processes, including the regulation of mood and emotional well-being. When tasting a chili pepper, the activation of TRPV1 channels by CAP results in the sensation of spiciness and the release of neurotransmitters, contributing to chilis’ pungent flavor. Intriguingly, CAP, a ligand for TRPV1, has been shown to have potential antidepressant and anxiolytic effects, although this aspect of TRPV1 function has received limited attention until recently.

Thus, mice given the TRPV1 antagonist α-spinasterol (1 or 2 mg/kg, i.p.) show decreased immobility during the FST. This effect is increased when α-spinasterol and the TRPV1 antagonist capsazepine are administered together at a subthreshold dose. Similar to this, SB36679 (1 mg/kg), a TRPV1 antagonist, decreases immobility during the FST in mice subjected to stressful circumstances brought on by a mobility restraint regimen. The immobility of mice during the FST is also reduced by a TRPV1-desensitizing therapy with resiniferatoxin (3–5 doses of 0.1 mg/kg given subcutaneously [s.c.]). TRPV1-deficient mice also exhibit shorter FST immobility periods, supporting our observations [8]. Additionally, it has been revealed that a number of “candidate” endogenous TRPV1 ligands exist and have the potential to induce therapeutic effects against pathological situations, including behavioral disorders [9].

Numerous investigations have represented nanoemulsion as a potential drug delivery carrier for phytochemicals and bioactive compounds, due to their tremendous advantages in solubility enhancement, spontaneous emulsification, and thermodynamic and kinetic stability over ordinary emulsions [10,11,12,13,14]. Various nanomaterials-based drug delivery carriers, such as nanoliposomes [15]; polymeric micelles [16]; solid lipid nanoparticles [17,18,19]; polymeric nanoparticles [20,21,22,23]; nanostructured lipid carriers [17,24,25]; and silver nanoparticles [26] of CAP have been studied in literature for the enhancement of CAP’s drug delivery potential, skin penetration, therapeutic potential, and pharmacokinetic profile. A nanoemulsion formulation of CAP has also been studied to enhance its skin penetration potential [27]. However, the antidepressant activity of CAP-loaded nanoemulsion has not been studied yet in the literature. Therefore, this research paper aims to evaluate the antidepressant activity of CAP-loaded nanoemulsion in mice affected by NC withdrawal-induced depression.

## 2. Materials and Methods

### 2.1. Drugs and Chemicals

Fluoxetine (FLX) was procured from Cadila pharmaceuticals Ltd. (Ahmedabad, India). CAP; polyethylene glycol 400 (PEG 400); bovine serum albumin; monobasic phosphate; dibasic phosphate; trichloroacetic acid; 5,5-dithio-bis-(2-nitrobenzoic acid) (DTNB); sodium pyrophosphate; phenazinium methosulphate; nitro blue tetrazolium; nicotinamide adenine dinucleotide (NADH); butanol; and NC hydrogen tartrate were obtained from Sigma Aldrich Ltd. (Mumbai, India). Serotonin standard and dopamine standard were obtained from E-Merck (Mumbai, India).

### 2.2. Formulation of the Nanoemulsion of CAP

CAP-loaded nanoemulsion was prepared using the ultrasonication method [28]. As part of mixture A, various weights of CAP were dissolved in various amounts of ethyl oleate and vortexed for 10 min. Next, 10 mL of absolute ethanol were mixed with different amounts of PEG 400 for 10 min (mixture B). Additionally, mixture A and mixture B were combined into a homogenous solution (mixture C) and vortexed for 15 min. To create nanoemulsion, mixture C was titrated dropwise with 40 mL of triple-distilled water, containing different amounts of zein, at 1200 rpm for 30 min. The resulting nanoemulsion was further processed using rotary evaporation to remove excess ethanol from the emulsion. Further, emulsions were ultra-sonicated using a probe sonicator to get a uniform micellar solution.

### 2.3. Evaluation of the Nanoemulsion

#### 2.3.1. Determination of Droplet Size, Polydispersity Index (PDI), and Zeta Potential

The mean or average size of the droplet, the zeta potential, and the PDI of the nanoemulsion were quantified using the quasi-elastic light scattering method, utilizing a Zetasizer or particle size analyzer (Malvern instrument ZA-90, Malvern, UK) at a scattering angle of 90°. For the estimation of those parameters, dilutions of the sample were done with milli-Q water in a test tube and, using a disposable cuvette, the diluted samples were measured in triplicate at 25 ± 1 °C. For the zeta potential, a zeta cell was used [29,30].

#### 2.3.2. Entrapment Efficiency (EE)

EE was performed to determine the drug amount entrapped in nanoemulsion, which helps in the release study of the drug. The amount of entrapped drug was evaluated using the centrifugation method, in which the free drug was separated from the nanoemulsion using a centrifuge. In this method, the prepared nanoemulsion was centrifuged at 15,000 rpm for 25 min with a maintained temperature of 4 °C, to obtain a supernatant layer. The collected supernatant layer was then diluted with the appropriate solvent and, using a reported high-performance liquid chromatography method, the amount of free drug was estimated from the diluted sample [31]. Using the following formula, the EE was calculated [32]:

(1) $$\%EE=\frac{Wt-Wu}{Wt}\times 100.$$

Here,

Wt = total amount of drug added in the formulation,

Wu = unbound drug estimated from the layer of the supernatant.

#### 2.3.3. Production Yield

The production yield of the nanoemulsion was determined by accurately measuring the initial weight of the raw materials and the final weight of the nanoemulsion recovered, then performing calculations using the formula [33]:

(2) $$Production\;yield=\frac{Practical\;mass\;of\;NE}{Theoretical\;mass\;of\;NE}$$

### 2.4. In Vivo Study

#### 2.4.1. Experimental Animals

Male BALB/c mice (2–3 months, 25–35 g) were used in this study. The animals were well cared for in a normal research laboratory environment, with free access to water and a 12 h natural light/dark cycle, as well as a noise level of less than 85 decibels. The Institutional Animal Ethics Committee has given its approval to the experimental protocol (IEAC) (Project proposal No. JSSAHER/CPT/IAEC/079/2021; approval date: 27 November 2021). The animals were cared for in accordance with the CPCSEA’s guiding principles, as well as those of the Ministry of Environment and Forests of India.

#### 2.4.2. Induction of Depression in Mice

NC was dissolved with 0.9% NaCl, and 2 mg/kg b.w was administered through the s.c. route for 7 days, thrice a day, and withdrawn on the 8th day; and the treatment with CAP at 1 mg/kg and 3 mg/kg was administered parallelly through the oral route (p.o.) for 14 days, and the behavioral tests were performed on 0th day, 7th day, and 14th day. Animals were euthanized on the 15th day to collect the brain samples for further biochemical assay [34]. The study plan is depicted in Table 1.

### 2.5. Behavioral Assessment

Animals were placed in the behavioral analysis room for about 1 h before experimenting.

#### 2.5.1. Tail Suspension Test (TST)

The TST entails hanging mice by their tails above the ground. Every mouse has its own three-walled rectangular compartment in which it is suspended. The mouse is suspended in the middle of this compartment, which is wide enough and deep enough to prevent the mouse from colliding with the walls. The distance between the mouse’s nose and the apparatus floor is approximately 20–25 cm in this environment. The animals were raised into the air using a metal hook and a piece of adhesive tape that was wrapped around their tails until it reached a height of around 15 cm. There is a video recording unit in use. Live scoring would be difficult since this test normally includes several animals being measured at the same time. The recording will begin after all the tapes have been applied. The free end of the tape is affixed to the suspension bar or shelf in a counterbalanced arrangement between treatment groups, and the animals are then suspended. It’s crucial to suspend the mice in a manner that does not obstruct the camera’s view during the entire TST session, as this would hinder the ability to monitor their behaviors throughout the entire duration. At the end of the session, which typically lasts for six minutes, the animals are placed back in their home cages. The tape is then removed from each tail with great care, gently peeling it off [34].

#### 2.5.2. Force Swim Test (FST)

The FST is a mouse behavioral test that evaluates the effectiveness of both existing and novel antidepressant compounds. Mice were placed in a water-filled, impenetrable transparent tank, and their mobility activity linked to escape was measured. The tanks should be filled with water that is set to room temperature (23–25 °C) and filled to the specified level, which is marked on the tank sides. When all the mice have been placed in the tanks, the stopwatch will begin counting down. For mice, a six-minute test is normal. Mice float easily in water, but they also make slight motions to keep their bodies balanced and their heads above the water. These actions are not an effort to flee, and they should not be counted as mobility. Also, even though mice are effectively immobile after a single bout of mobility, they can still float in the water due to momentum. These actions should also not be counted as mobility. The amount of time each mouse spends on the move is recorded. The immobility time is calculated by subtracting the cumulative sum of mobility time from the 240 s of test time [35].

#### 2.5.3. Open Field Test (OFT)

The mice’s locomotor activity during the OFT [36] was measured using an opaque plastic box with the dimensions 30 × 30 × 30 cm^3^ that was divided into 16 (4 × 4 cm^2^) identical sectors measuring 7.5 × 7.5 cm^2^. The central sector, which consists of 4 central squares and was divided into 2 × 2 cm^2^ and the rest squares, was created by dividing the periphery group into major sectors of the center. The mice were placed separately in the box with faded light for around 10 min, and the area was cleaned with 70% ethanol between each test. Based on the mouse’s freedom to roam around in a squared region between 43.2 cm and 43.2 cm for 5 min, the mouse’s locomotion was observed. The extrapolatory activity was measured by the number of squares crossed, the amount of freezing time, and the amount of time spent in the corner.

### 2.6. Biochemical Parameters

#### 2.6.1. Evaluation of Superoxide Dismutase (SOD) and Reduced Glutathione (GSH)

Whole brain homogenates were prepared using a 10% phosphate buffer solution. Firstly, brains were carefully dissected and immediately placed in a pre-cooled phosphate buffer solution. The brain tissue was then homogenized using a homogenizer to ensure uniform consistency. The resulting homogenate was maintained as a 10% solution. After homogenization, the samples were subjected to centrifugation at 4000 rpm for 10 min, to separate the supernatant from the remaining cellular components. The supernatant was collected and used for subsequent assessments of antioxidant properties. This procedure ensured that the brain homogenates were prepared in a consistent and standardized manner, allowing for accurate measurement of antioxidant markers [37].

#### 2.6.2. Total Protein

Lowry’s approach was used to calculate protein content. After preparing the tissue homogenate, 0.1 mL was added to the test tube. The test tube was then filled with distilled water to a volume of 1 mL. Blank was preserved in the form of water. Reagent A, which is made up of 2 percent sodium carbonate, was prepared by adding 2 g of sodium carbonate to 100 mL of 0.1 N sodium hydroxide solution, while reagent B is made up of 0.5 percent copper sulphate solution mixed with 1 percent potassium tartrate. Reagent C is made up of 50 mL of reagent A mixed with 1 mL of reagent B. Reagent D, which was pre-prepared, was identified as the Folin–Ciocalteau reagent. Subsequently, 5 mL of reagent C was added to the blank solution, and this mixture was left at room temperature for 10 min in low-intensity light. The absorbance values were measured at 660 nm. From a concentration of 20 to 100 μg/mL, normal bovine serum albumin was plotted as a standard [38].

#### 2.6.3. Superoxide Dismutase (SOD)

The assay for SOD was performed by adding 0.025 mL of phenonium methosulphate, which has a molecular weight of 186 µM and a concentration of 5.7 mg per 100 mL of water, to 0.05 mL of tissue homogenate. Next, 0.025 mL of sodium pyrophosphate buffer (0.3 mL) at a pH of 8.3 was added. Then, 0.0075 mL of NADH was added to 780 μM of pH 8.3 buffer to start the process; after an incubation period of one and a half minutes at a temperature of 30 °C, 0.25 mL of glacial acetic acid was added to stop the reaction. The mixture was then given 2.0 mL of n-butanol, followed by centrifugation, where “n-butanol” was used as a blank. At 560 nm, the absorbance was measured [39].

##### Reagent Preparation

Sodium pyrophosphate buffer of 0.025 M is prepared by dissolving 0.66475 g in 100 mL water, and the pH is adjusted to 8.3. Phenazinium methosulphate (PMS) is prepared by dissolving 2.85 mg PMS in 10 mL of water. NBT is prepared in a buffer (pH 8.3); 3 mg of NBT in 10 mL of sodium pyrophosphate buffer. NADH is also prepared in a buffer (pH 8.3); 6.19393 mg of NADH in 10 mL of sodium pyrophosphate buffer. Butanol alone serves as a blank solution.

#### 2.6.4. Reduced Glutathione (GSH)

After mixing 0.25 mL of the tissue homogenate with 5% trichloroacetic acid, GSH was measured. After that, the solution was centrifuged for about 10 min at 4000 rpm. Next, 0.2 M of phosphate buffer and 0.5 mL of DTNB were added and thoroughly combined. At 412 nm, the spectra were measured [40].

##### Reagent Preparation

In order to create a standard stock solution, 5 mg of GSH was dissolved in 100 mL distilled water. TCA 5% was prepared by dissolving 5 g of TCA in 100 mL water. Phosphate buffer with a pH of 8.0 is prepared using a mixture of monobasic and dibasic phosphate (monobasic phosphate (0.2 M)–2.839 g in 100 mL water; dibasic phosphate (0.2 M)–2.399 g in 100 mL water). To prepare the phosphate buffer and adjust the pH to 8.0, 5.3 mL of monobasic phosphate solution and 94.7 mL of dibasic phosphate solution is mixed. DTNB is prepared by dissolving 24 mg of DTNB in 100 mL of phosphate buffer. Blank solution: 0.25 mL of 5% TCA + 0.25 mL of phosphate buffer + 0.5 mL of DTNB.

#### 2.6.5. Lipid Peroxidation (MDA)

TBARS was measured using 0.2 mL of the homogenate of the brain, and then the saline solution was added up to 0.8 mL. Further, 1 mL of trichloroacetic acid and 1 mL of hydrochloric acid were added to the homogenate solution and centrifuged for about 2000 rpm for 10 min. The absorbance of the supernatant was determined at 532 nm. Phosphate buffer was taken as a blank solution.

### 2.7. Statistical Analysis

The behavioral analysis was performed through two-way ANOVA, tailed by the post hoc Bonferroni test; the antioxidant markers were analyzed through one-way ANOVA with a post hoc Tukey test. All the statistics were expressed in mean ± standard error of mean (SEM).

## 3. Results

The current study has demonstrated the CAP-loaded nanoemulsion as an antidepressant agent. However, behavior and biochemical tests have been performed. The CAP-loaded nanoemulsion has shown activity in suppressing depression, like that of the standard FLX.

### 3.1. Characterization of CAP Nanoemulsion

#### 3.1.1. Determination of Droplet Size and PDI

**Figure 1:**
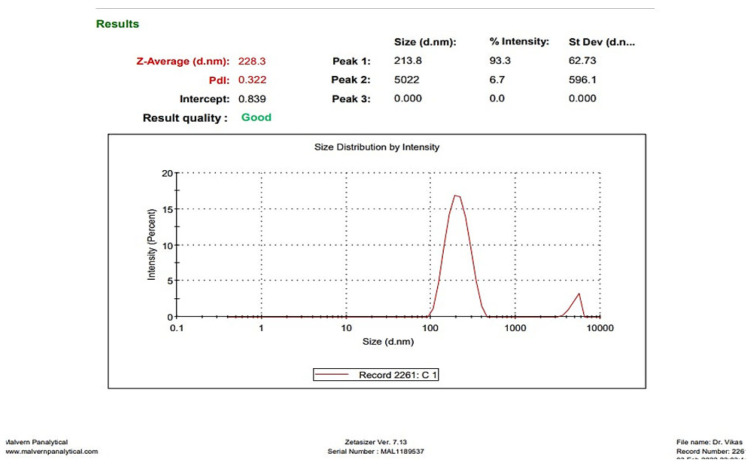
The droplet size of CAP-loaded nanoemulsion, measured using the DLS technique.

The droplet size and PDI of CAP-loaded nanoemulsion were measured using the DLS technique. The representative image is presented in Figure 1. The droplet size of developed nanoemulsion was measured to be 228.3 nm, with a PDI of 0.322.

#### 3.1.2. Determination of Zeta Potential

**Figure 2:**
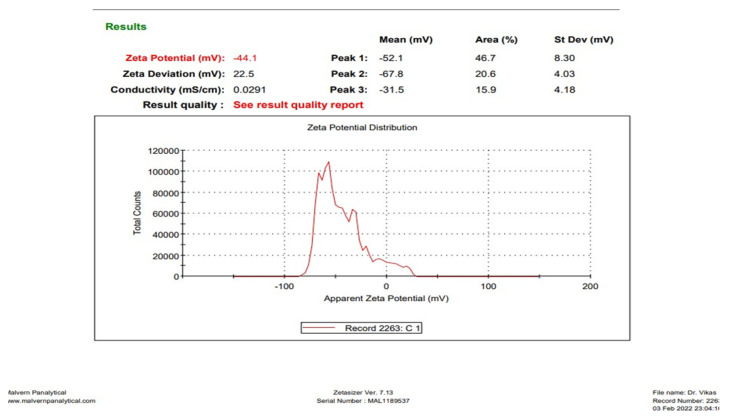
The zeta potential of CAP-loaded nanoemulsion, measured using the DLS technique.

The zeta potential of CAP-loaded nanoemulsion was also measured using the DLS technique. The representative image is presented in Figure 2. The zeta potential of CAP nanoemulsion was measured to be −44.1 mV.

#### 3.1.3. EE

The EE of the CAP-loaded nanoemulsion was found to be 86.4%. With an increase in ethyl oleate, the EE was found to also increase.

#### 3.1.4. Product Yield

The product yield of CAP-loaded nanoemulsion was found to be 64 ± 2%. This was estimated using the theoretical and actual weights of the product acquired.

### 3.2. Behavioral Assessments of NWM

#### 3.2.1. TST

**Figure 3:**
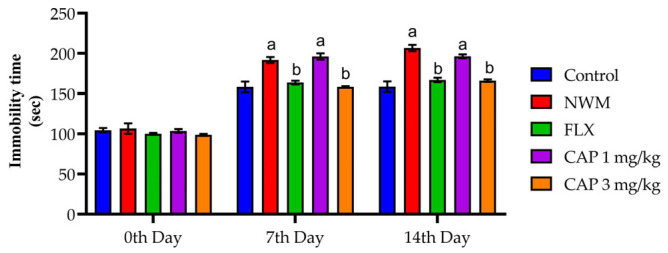
The effect of CAP-loaded nanoemulsion on TST immobility in NWM. Data were expressed as mean ± standard error of mean, *n* = 6. Data were analyzed through two-way ANOVA, followed by Bonferroni’s post-t-test. ^a^ *p* < 0.05 versus control group. ^b^ *p* < 0.05 versus NWM group.

Elevated mobility was seen in the NMW group on the 14th day due to NC withdrawal, with a significance of *p* < 0.05 compared to the control group. CAP 3 mg/kg has shown a significant (*p* < 0.05) decrease in immobility compared that of the NMW group, as shown in Figure 3.

#### 3.2.2. FST

**Figure 4:**
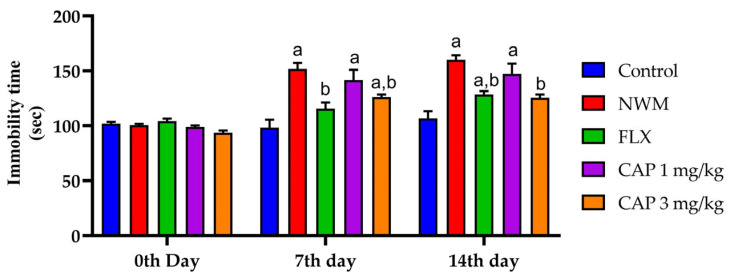
The effect of CAP-loaded nanoemulsion on FST immobility in NWM. Data were expressed as mean ± standard error of mean, *n* = 6. Data were analyzed through two-way ANOVA, followed by Bonferroni’s post-t-test. ^a^ *p* < 0.05 versus control group. ^b^ *p* < 0.05 versus NWM group.

During the FST, the immobility time of the NMW group was considerably increased on the 7th and 14th day (*p* < 0.05) as compared with the control group. Similarly, CAP 3 mg/kg showed a considerable (*p* < 0.05) decrease in immobility time compared to the NMW group on both the 7th and the 14th day, as shown in Figure 4.

#### 3.2.3. OFT

**Figure 5:**
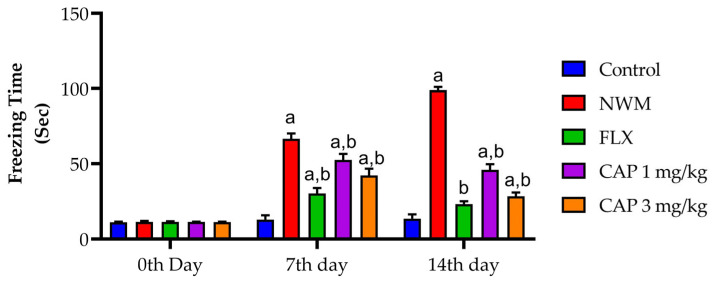
The effect of CAP-loaded nanoemulsion on freezing time during the open field test on NWM. Data were expressed as mean ± standard error of mean, *n* = 6. Data were analyzed through two-way ANOVA, followed by Bonferroni’s post-t-test. ^a^ *p* < 0.05 versus control group. ^b^ *p* < 0.05 versus NWM group.

**Figure 6:**
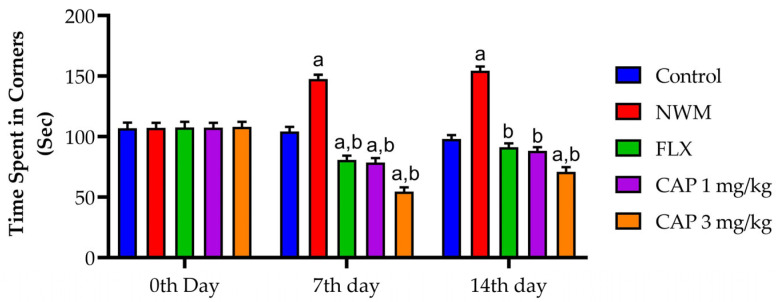
The effect of CAP-loaded nanoemulsion on time spent in the corner during the OFT on NWM. Data were expressed as mean ± standard error of mean, *n* = 6. Data were analyzed through two-way ANOVA, followed by Bonferroni’s post-t-test. ^a^ *p* < 0.05 versus control group. ^b^ *p* < 0.05 versus NWM group.

During the OFT, the evaluation of NWM was done in two sets: freezing time and time spent in the corner. The immobile (freezing) time of the NWM group showed a considerable increase (*p* < 0.05) in the time spent immobile compared to that of the control group. Test 2 showed a considerable (*p* < 0.05) decrease in time spent immobile during the OFT compared to that of the standard FLX. The time spent in the corner was considerably (*p* < 0.05) longer in the NMW group when compared with the control group. Test 2 showed a considerable (*p* < 0.05) decrease in the time spent in corners during the OFT compared to that of the NMW group, as shown in Figure 5 and Figure 6.

### 3.3. Antioxidant Markers

#### 3.3.1. SOD

**Figure 7:**
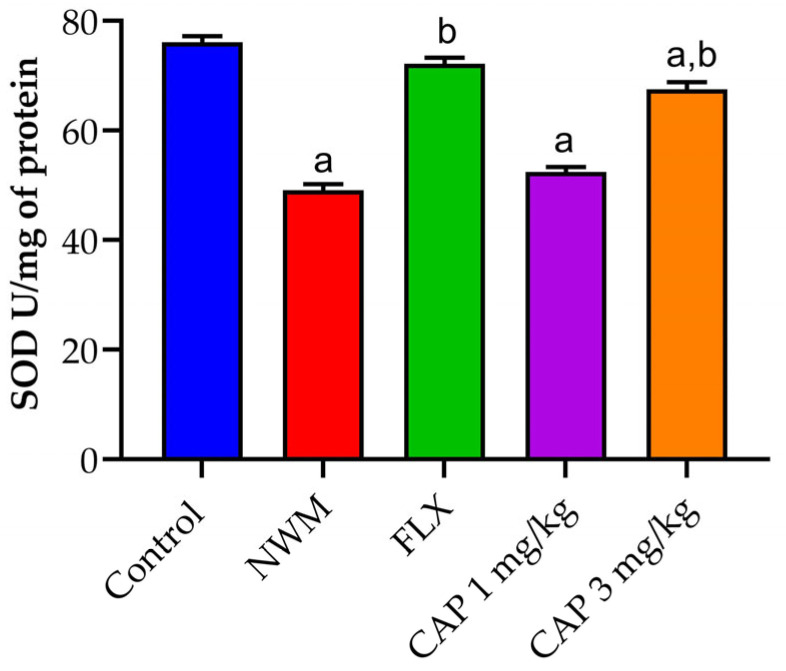
The effect of CAP-loaded nanoemulsion on superoxide dismutase activity in hippocampus samples of NWM. Data were expressed as mean ± standard error of mean, *n* = 3. Data were analyzed through one-way ANOVA, followed by the Tukey test. ^a^ *p* < 0.05 versus control group. ^b^ *p* < 0.05 versus NWM group.

The induction of depression in NWM considerably (*p* < 0.05) reduced SOD activity in the hippocampus samples of the NMW group as compared to the control group. CAP 3 mg/kg showed similar activity to that of FLX 20 mg/kg, the standard group, as shown in Figure 7.

#### 3.3.2. GSH

**Figure 8:**
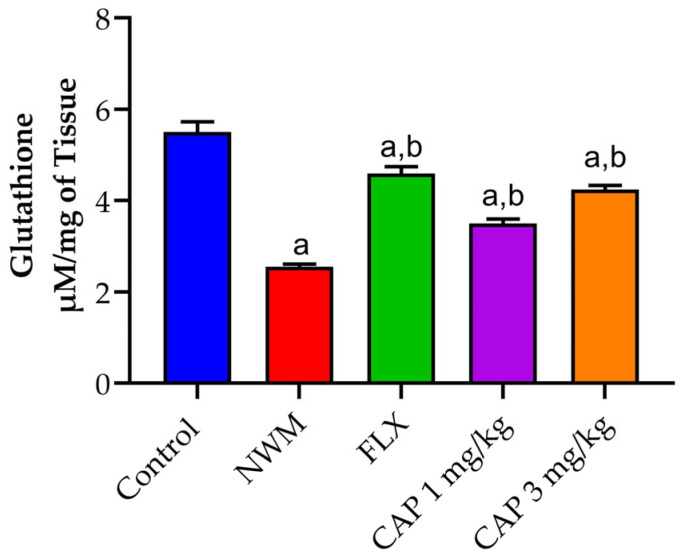
The effect of CAP-loaded nanoemulsion on GSH activity in hippocampus samples of NWM. Data were expressed as mean ± standard error of mean, *n* = 3. Data were analyzed through one-way ANOVA, followed by the Tukey test. ^a^ *p* < 0.05 versus control group. ^b^ *p* < 0.05 versus NWM group.

The induction of depression in NWM considerably (*p* < 0.05) reduced GSH activity in the hippocampus samples of the NMW group as compared to the control group. CAP 3 mg/kg showed similar activity to that of FLX 20 mg/kg, the standard group, as shown in Figure 8.

#### 3.3.3. Lipid Peroxidation

**Figure 9:**
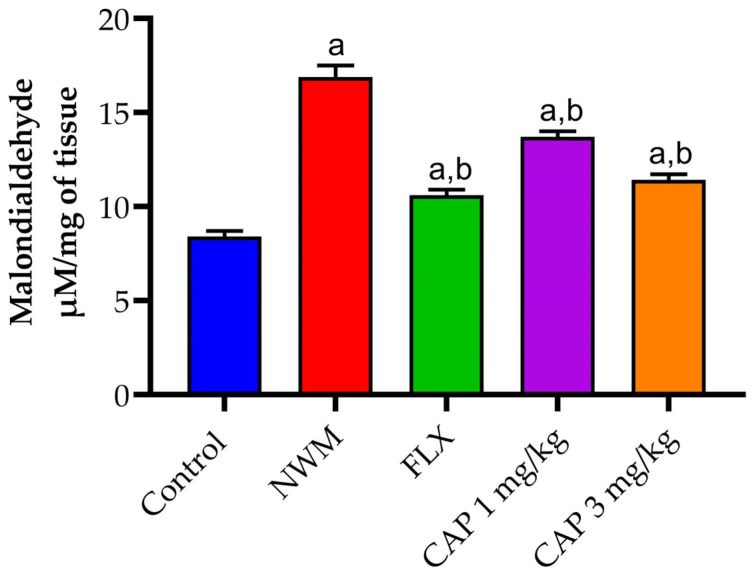
The effect of CAP-loaded nanoemulsion on malondialdehyde activity in hippocampus samples of NWM. Data were expressed as mean ± standard error of mean, *n* = 3. Data were analyzed through one-way ANOVA, followed by the Tukey test. ^a^ *p* < 0.05 versus control group. ^b^ *p* < 0.05 versus NWM group.

The induction of depression in NWM considerably (*p* < 0.05) increased malondialdehyde activity in the hippocampus samples of the NWM group as compared to the control group. CAP 3 mg/kg showed activity similar to that of FLX 20 mg/kg, the standard group, as shown in Figure 9.

## 4. Discussion

In this study, the nanoemulsion formulation of CAP was developed using an ultrasonication technique and characterized for various physicochemical parameters [28]. The results of physicochemical characterization suggested the stable formation of CAP-loaded nanoemulsion. After physicochemical characterization, the intended formulation was subjected to in vivo studies using a mouse model.

According to reports, depression is one of the most common psychiatric conditions and is thought to be the second largest cause of neurological disorders and impairment globally [2]. Tail suspension is one of the main stressors used to evaluate depressive-like behavior in mice, in this case caused by depression that was induced by nicotine withdrawal [34]. The mice’s immobility was measured during the assessment over a predetermined interval of time.

It was observed that the group treated with CAP-loaded nanoemulsion (3 mg/kg) exhibited a level of immobility comparable to that of the group administered with FLX (20 mg/kg). Importantly, both of these treatment groups showed a reduced level of immobility in comparison to the NWM group. This reduction in immobility can be attributed to potential effects on the HPA axis. It is plausible that the CAP-loaded nanoemulsion and FLX treatments may have desensitized or stabilized the HPA axis in response to physical stressors. In contrast, the NWM group displayed an increase in immobility during the evaluation period, which suggests a different response to the stressors introduced in the experiment [2,41].

One of the main stressors used to assess depressive-like behavior in mice afflicted with NC withdrawal-induced depression is the FST [35]. The assessment included an examination of the extent of immobility exhibited by the mice during the test period as a component of the evaluation process. Notably, the NWM group demonstrated a notable increase in immobility over time. In contrast, the group treated with CAP-loaded nanoemulsion (3 mg/kg) displayed a level of immobility equivalent to that of the group treated with FLX (20 mg/kg), and this level of immobility was lower compared to the NWM group. This observation suggests a potential relationship with the HPA axis [35].

One important depression behavioral assay is OFT [36]. The immobility time and time spent in corners were used to evaluate the characteristics of mice in the open field. When compared to the control group, the NWM group had a higher percentage of time spent in the corner. The experimental assessment revealed a reduction in both immobility and the amount of time spent in the corner in the group that received CAP-loaded nanoemulsion (3 mg/kg), and this effect was equivalent to the group administered with standard FLX (20 mg/kg). To elaborate, the study found that the group treated with CAP-loaded nanoemulsion exhibited a decrease in both the amount of time spent in the corner and the duration of immobility, and the reduction in immobility duration was comparable to the group receiving the standard FLX (20 mg/kg). This suggests that CAP-loaded nanoemulsion may have a similar effect to FLX in reducing immobility, indicating a potential therapeutic benefit in the context of this study [36].

In order to assess the antioxidant effect of NWM-induced depression, endogenous antioxidant enzyme levels in the brain were estimated [37]. SOD, an endogenous antioxidant enzyme, was present at lower levels in the NWM group. SOD plays a significant part in antioxidant properties [42]. The SOD enzyme family is crucial for preserving healthy physiological conditions and dealing with stress in living cells, and SOD is necessary in order to safeguard the biological integrity of cells and tissues from the damaging effects of superoxide free radicals [43]. With the whole brain sample of mice treated with CAP-loaded nanoemulsion (3 mg/kg), the same amount of SOD increase was seen upon the induction of NWM as would occur with standard FLX (20 mg/kg).

Endogenous antioxidant enzyme levels in the brain were calculated to determine the antioxidant effect of NWM-induced depression. The detoxification of reactive oxygen species (ROS) in the brain is greatly aided by glutathione. The recognized capacity or resistance to ROS of different types of brain cells can depend on changes in antioxidant activity or small molecular weight concentrations, as well as the accessibility of precursors for glutathione production and NADPH regeneration [44]. Levels of endogenous antioxidant GSH were found to be reduced under physiological and pathophysiological conditions. It was discovered that NWM induction resulted in a significant decrease in GSH levels in comparison to standard FLX (20 mg/kg), although the CAP-loaded nanoemulsion (3 mg/kg) group showed the same insightful activity as the standard.

Lipid peroxidation is a sequence of reactions involving free radicals that, once started, results in the oxidative degradation of polyunsaturated lipids [45]. Because of its high polyunsaturated content and high brain oxygen intake, the central nervous system is susceptible to lipid peroxidation. It also lacks antioxidant enzymes in a significant amount [46,47,48,49]. Plasma MDA levels were found to be higher in people who suffer from depression. Because of the oxidative stress induced by nicotine withdrawal, MDA levels in the NWM group were elevated. The CAP-loaded nanoemulsion (3 mg/kg) has shown the same insightful activity as the standard [50].

In summary, our findings indicate that CAP-loaded nanoemulsion has the potential to alleviate depressive-like behaviors, and to modulate antioxidant enzyme levels in a mouse model of NC withdrawal-induced depression. The results of our study suggest that CAP-loaded nanoemulsion may have therapeutic benefits in addressing the complex interplay of depressive behavior and oxidative stress associated with NC withdrawal. These promising outcomes warrant further investigation and hold the potential for the development of novel treatments for NC withdrawal-induced depression. Moreover, this study contributes to the broader understanding of the mechanisms involved in depression and the potential of nanoemulsion-based drug delivery systems in psychiatric research. Future research should focus on elucidating the specific pathways through which CAP-loaded nanoemulsion exerts its effects, and explore potential clinical applications.

## 5. Conclusions

Based on the above results obtained from the behavioral and biochemical evaluations, CAP-loaded nanoemulsion at 3 mg/kg has combated depression in the mice afflicted with NC withdrawal-induced depression, and may be used as a supplementary treatment. However, the mechanisms behind CAP-loaded nanoemulsion’s effects on depression need to be further evaluated and confirmed by molecular level studies.

## Figures and Tables

**Table 1:** Grouping of animals and dosage schedule of nicotine (NC) withdrawal model.

S. No.	Group (*n* = 6)	Treatment
1	Control	NC (2 mg/kg [s.c.] thrice a day up to 7 days + CMC 1% [10 mL/kg p.o] once in a day up to 14 days) was administered
2	NWM	NC (2 mg/kg [s.c.] thrice a day up to 7 days + CMC 1% [10 mL/kg p.o] once in a day up to 14 days) was administered
3	FLX	NC (2 mg/kg [s.c.] thrice a day up to 7 days + FLX [20 mg/kg p.o] once in a day up to 14 days) was administered
4	CAP 1 mg/kg	NC (2 mg/kg [s.c.] thrice a day up to 7 days + CAP low dose [1 mg/kg p.o] once in a day up to 14 days) was administered
5	CAP 3 mg/kg	NC (2 mg/kg [s.c.] thrice a day up to 7 days + CAP [3 mg/kg p.o] once in a day up to 14 days) was administered

Abbreviations: NWM: nicotine withdrawal model; FLX: fluoxetine; CAP: capsaicin.

## Data Availability

The data presented in this study are available on reasonable request from the corresponding author. The data are not publicly available due to privacy and ethical restrictions.
